# Frequency and Fitness Consequences of Bacteriophage Φ6 Host Range Mutations

**DOI:** 10.1371/journal.pone.0113078

**Published:** 2014-11-19

**Authors:** Brian E. Ford, Bruce Sun, James Carpino, Elizabeth S. Chapler, Jane Ching, Yoon Choi, Kevin Jhun, Jung D. Kim, Gregory G. Lallos, Rachelle Morgenstern, Shalini Singh, Sai Theja, John J. Dennehy

**Affiliations:** 1 Biology Department, Queens College of the City University of New York, New York, New York, United States of America; 2 The Graduate Center of the City University of New York, New York, New York, United States of America; Centro Nacional de Biotecnologia - CSIC, Spain

## Abstract

Viruses readily mutate and gain the ability to infect novel hosts, but few data are available regarding the number of possible host range-expanding mutations allowing infection of any given novel host, and the fitness consequences of these mutations on original and novel hosts. To gain insight into the process of host range expansion, we isolated and sequenced 69 independent mutants of the dsRNA bacteriophage Φ6 able to infect the novel host, *Pseudomonas pseudoalcaligenes.* In total, we found at least 17 unique suites of mutations among these 69 mutants. We assayed fitness for 13 of 17 mutant genotypes on *P. pseudoalcaligenes* and the standard laboratory host, *P. phaseolicola*. Mutants exhibited significantly lower fitnesses on *P. pseudoalcaligenes* compared to *P. phaseolicola*. Furthermore, 12 of the 13 assayed mutants showed reduced fitness on *P. phaseolicola* compared to wildtype Φ6, confirming the prevalence of antagonistic pleiotropy during host range expansion. Further experiments revealed that the mechanistic basis of these fitness differences was likely variation in host attachment ability. In addition, using computational protein modeling, we show that host-range expanding mutations occurred in hotspots on the surface of the phage's host attachment protein opposite a putative hydrophobic anchoring domain.

## Introduction

After a long period of steady decline, mortality due to infectious disease increased over the past several decades, largely because of the emergence of new infectious diseases including HIV [1], [2]. Of these new diseases, a disproportionate number have been viruses [3], [4]. Because of their high mutation rates and vast population sizes, viruses have higher probabilities of acquiring the requisite mutation(s) allowing infection of novel hosts than do other types of pathogens [5]. A common fear is that a highly transmissible and virulent virus will spread pandemically among humans, causing widespread mortality and economic damage. Thus, there is a strong motivation to understand and predict virus emergence.

Virus emergence is a two-step process. A virus first mutates to gain the ability to infect a new host, and then fully emerges by achieving positive population growth on that host via adaptation [6]. Theoretical modeling has shown that emergence probabilities are highly sensitive towards the type of mutation(s) required to productively infect a novel host [7]. Emergence events requiring single nucleotide substitutions are far more likely to occur than those that require several simultaneous point mutations or recombination [8]. While mutations altering virus host specificity can involve large-scale genomic rearrangements, most virus host shifts likely entail the modification of a small number of virus receptor amino acid residues [9]. In fact, single nucleotide substitutions are often sufficient to expand a virus's host range [10]. If this mechanism of host range expansion were common, the number of host range expanding mutations and their frequency of appearance would be important parameters governing the probability of emergence of a potential human pathogen.

Few studies have systematically determined the type, number, frequency, and fitness consequences of host range expanding mutations for any particular virus-host combination [11]. Such data can aid the parameterization of evolutionary ecological model of virus emergence. Factoring in other parameters, such as transmission rates and population densities, may allow quantitative predictions of the likelihood a particular virus is able to emerge on a new host. This type of prioritization is critical before allocating resources to interdict potential pathogenic viruses before they emerge.

Here we use an experimental model system, the bacteriophage (phage) Φ6, to determine number, frequency, fitness and structural consequences of mutations allowing infection of a novel host. Phage Φ6 (family Cystoviridae) is a dsRNA virus with a tripartite genome divided into Small (2,948 bp), Medium (4,061 bp) and Large (6,374 bp) segments [12]–[14]. Mutations allowing Φ6 to infect novel hosts have been localized to the gene encoding the P3 protein on the Medium segment [11], [15].

Two previous studies have systematically examined Φ6 host range expansion [11], [15]. Duffy et al. isolated 10 Φ6 host range mutants on each of three different *Pseudomonas* host strains including *Pseudomonas pseudoalcaligenes* [15]. Genetic sequencing revealed that all mutations occurred in the P3 attachment protein. Moreover, the authors reported that 7 of the 9 host range mutations imposed a fitness cost on the canonical host, indicative of antagonistic pleiotropy. Ferris et al. isolated 40 Φ6 host range mutants on *P. glycinea*, of which 16 contained novel mutations [11]. The authors used a statistical approach to predict the existence of a further 39 mutations that were missed by their screen. In addition, they observed broad fitness costs on the canonical host in agreement with Duffy et al. [11], [15].

Our study builds on each of these earlier studies in order to present a more complete picture of host range expansion in Φ6. We isolated 69 independent Φ6 mutants able to infect the novel host, *P. pseudoalcaligenes*, and sequenced the entire P3 gene for each of them in order to determine the number, location, and frequency of host range expanding genotypes. For a subset of unique mutant genotypes, we quantified plaque size on *P. pseudoalcaligenes*, and reproductive capacity (fitness) and host attachment rate on both the canonical host *P. phaseolicola* and the novel host, *P. pseudoalcaligenes*. Finally, we used protein structural modeling to predict the effects of host range mutations on P3 attachment protein structure. Our work comes to qualitatively different conclusions than earlier work, namely that: 1) the coupon collector's model, as currently construed, cannot successfully predict the number of potential host range mutations; 2) there are fewer than expected mutations allowing host range expansion; and 3) fine-grained host infectibility cannot be accurately predicted by phylogeny. Furthermore, we show that Φ6 host range mutations usually occur in hotspots on the face of the P3 attachment protein opposite a hydrophobic anchoring domain. We propose that mutations on this surface allow Φ6 to bind novel host receptors.

## Methods and Materials

### Study Organisms and Culture Conditions

Cystovirus Φ6 used in these experiments is a descendant of the strain originally isolated from bean straw in 1973 [16]. Φ6's host of isolation was the Gram-negative bacterium, *P. syringae* pathovar *phaseolicola* (ATCC # 21781; hereafter PP) [16]. In our study, we used a nonpermissive host, *P. pseudoalcaligenes* East River isolate A (hereafter ERA), to isolate Φ6 host range mutants (HRMs). The ERA receptor to which Φ6 binds has not been determined, but is likely the ERA pili. Two other nonpermissive hosts, P. *syringae* pv. *tomato* (hereafter TOM) and P. *syringae* pv. *atrofaciens* (hereafter ATRO), were used in some assays. All bacteria and virus stocks were obtained from Paul Turner, Yale University, New Haven, CT.

All phages and bacteria were propagated in lysogeny broth (LB: 10 g NaCl, 10 g Bacto tryptone, and 5 g Bacto yeast extract per liter of water) at pH 7. Bacterial cultures were initiated by transferring a single colony from a streak plate into 10 mL LB in a sterile 50 mL flask capped with a 20 mL beaker. Culture flasks were incubated with shaking (120 rpm) at 25°C for 18 hours, allowing bacteria to attain stationary-phase density (∼6×10^9^ cells mL^−1^).

### Virus Stock Preparation

High-titer phage lysates were prepared by adding 1 µL stock lysate and bacteria (200 µL of PP/ATRO/TOM or 20 µL ERA) to 3 mL top agar (LB with 0.7% Bacto agar; stored as liquid at 45°C, solidifies at 25°C), and pouring onto 35 mL bottom agar (LB with 1.5% Bacto agar) in a sterile Petri dish. After 24 hours at 25°C, the resulting plaques were harvested and resuspended in 4 mL of LB, followed by 10 min centrifugation at RCF  = 1400×g to pellet agar and bacterial debris. Bacteria-free lysates were obtained by filtering the supernatant through a 0.22 µm filter (Durapore; Millipore, Bedford, MA). Phage particles per mL in the lysates were quantified via serial dilution and titering. Plaques were counted on plates where 30–500 plaques were visible. The number of plaque forming units per mL (pfu mL^−1^) in the original lysate was obtained by multiplying the number of plaques times the dilution factor. Lysates were mixed 1∶1 (v/v) with 80% glycerol and were stored at −20°C.

### Host Range Mutant Frequency

The frequency of HRMs in a phage population was estimated by plating a known number of wildtype Φ6 on a lawn of a nonpermissive host and counting the resulting number of plaques. Each plaque represents the descendants of a single HRM in the parent population. To perform this assay, a single plaque was picked off a lawn of PP and placed in 1 mL LB. This mixture was serially diluted and plated on PP to estimate phage pfu mL^−1^. Subsequently, 10^7^ or (10^8^ for TOM) phages were plated on lawns of the nonpermissive hosts, ERA, TOM and ATRO. Typically, a Φ6 plaque contains ∼5×10^8^ pfu/mL so sufficient phage for plating were easily obtained [17]. Following 48 hrs growth, plaques were counted to estimate the number of spontaneous HRMs among the descendants of a single phage. This assay was repeated at least twenty times per nonpermissive host strain. Mutant frequency was calculated by dividing the number of plaques observed by the initial inocula. The resulting data were analyzed using a one-way analysis of variance (ANOVA) model with host as a factor. A Tukey-Kramer honest significant difference test was applied post-hoc to ascertain significant differences among mean mutant frequencies on each host type.

### Host Range Mutant Isolation

Each HRM was isolated independently to minimize bias due to a “jackpot effect” where multiple descendants of the same mutational event appear in a population [18]. A Φ6 lysate was serially diluted and plated on a PP lawn such that only a few widely spaced plaques appeared on the bacterial lawn. A single plaque was picked at random and placed in 1 mL LB. After vortexing, 100 µL of the plaque suspension was added to 20 µL ERA and 3 mL top agar and plated. Following 48 hrs growth, a single HRM plaque was picked from the ERA lawn and suspended in 500 µL LB. 10 µL from this solution was plated on an ERA lawn to obtain phage lysate for RNA extraction. 500 µL 80% glycerol was added to the remainder which was then stored at −20°C. This protocol was repeated 69 times to obtain 69 independent HRM isolates.

### RNA Extraction and Sequencing

To sequence the region of the Medium segment encoding the P3 protein, 3 mL phage lysate from each mutant was concentrated by centrifuging at RCF  = 100,000×g for 3 hrs at 4°C using a Beckman TL-100 ultracentrifuge. The supernatant was discarded and the pellet was resuspended in 150 µL nuclease-free water. RNA was extracted using a QIAamp Viral RNA Mini Kit (QIAGEN, Valencia, CA). Phage RNA was reverse transcribed using random hexamer primers and Superscript III reverse transcriptase (Life Technologies, Grand Island, NY), and the resulting cDNA was used as template for PCR. Three sets of oligonucleotide primers corresponding to bases 1298–2142, 2042–3052, and 2877–3873 of Φ6's Medium segment were used for the PCR amplification of the region encoding the P3 host attachment protein. PCR product was purified for sequencing using ExoSAP-It (Affymetrix, Santa Clara, CA). PCR product was sequenced in both directions with a minimum of 3-fold replication (6-fold coverage). Sequencing was performed at the DNA Analysis Facility on Science Hill at Yale University. Sequence data were analyzed using Geneious Pro Ver. 5.4 [19] and MEGA Ver. 5.05 [20]. Chromatograms were verified via MacVector Ver. 12.5.1 bioinformatics software.

### Mutant Characterization

We phenotypically characterized HRM genotypes by determining plaque size on ERA, and by assaying reproductive capacity (fitness) and attachment rates on ERA and PP. Of our unique mutant genotypes, we did not assay the three mutants whose mutations were not identified (see Table 1). Furthermore, we only assayed one mutant in situations where differences between genotypes were attributable to synonymous amino acid substitutions. Finally, for one mutant, stored frozen lysate degraded due to a freezer failure following sequencing, and viable phage could not be recovered for phenotypic characterization. In sum, we phenotypically characterized 13 of our 17 unique HRM genotypes.

**Table 1:** Phage φ6 Mutations Allowing Infection of Novel Host ERA.

Nucleic Acid (Amino Acid)	N	G22A (E8K)	A23G (E8G)	A23C (E8A)	G24T (E8D)	G24C (E8D)	T137C (F46S)	T138A (F46L)	T177C D59D)	A389G (Q130R)	T481C (S161P)	C896G (S299W)	C1588T (L530L)	A1661G (D554G)	A1661C (D554A)	C1663T (L555F)	Non P3
Single (n = 56)	20	▪															
	24		▪														
	4			□													
	3				□												
	1					□											
	1									▪							
	2													▪			
	1														□		
Double (n = 8)	1	▪					▪										
	1	▪								▪							
	1	▪												▪			
	1			□				□									
	3		▪							▪							
	1									▪		□					
Triple (n = 2)	1		▪							▪							
	1										▪					▪	
Unknown	3																▴
Total # Observed	69	23	28	5	3	1	1	1	1	7	1	1	1	3	1	1	3

In this table, the columns show the nucleotide and amino acid substitutions found it all 69 ERA host range mutants. Rows show unique genotypes. N is the number of mutants with a particular genotype. The last row shows the number of times a given mutation appears among all mutants. Transitions are indicated by closed squares, transversions with open squares, synonymous substitutions with open circles and unknown changes with a filled triangle.

### Plaque Size Estimates

Plaque sizes for 13 HRM genotypes were estimated from digital photographs of plaques formed on ERA. All LB plates used for plaque assays were poured at the same time and were weighed to maintain consistency. Each mutant's lysate was diluted and plated such that between 20 to 100 plaques formed on the ERA lawn after 48 hrs growth. Digital photographs were taken using a Kodak Gel Logic 440 digital imaging system. ImageJ software (NIH, Bethesda, MD; http://rsb.info.nih.gov/ij/) was used to estimate the total area of the plaque. For each genotype, at least 35 plaque size estimates were made across 3 plates.

### Mutant Fitness on Native and Novel Hosts

We assayed absolute fitness for 13 of our Φ6 mutants and the wildtype on native and novel hosts using traditional plating methods. Here 10^5^ phages were added to 3×10^8^ host cells in 10 mL LB and incubated at 25°C with rotary shaking (120 rpm) for 24 hrs. All assays were replicated 5x. Bacteria-free lysates were obtained by centrifuging 3 mL culture at RCF  = 2.75×g for 10 min to pellet bacterial debris, then passing the supernatant through a 0.22 µm filter. Phage particles per mL in the lysates were quantified via serial dilution and titering on host lawns. For each assayed mutant genotype, we estimated absolute fitness using the equation, 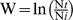, where N_i_ is the starting number of phage and N_t_ is the total number of progeny phage produced during the infection period.

### Attachment Rate Assays

The rate of attachment to native and novel hosts of 13 mutant HRM genotypes was measured using a centrifugation method. This method relies on the fact that, following centrifugation, attached phages are pelleted with host cells, while unattached phages remain in the supernatant. The decline of unattached phage over time is quantified to give the rate of phage attachment to host cells. In this assay, 10^3^ phages, which were titered on the same host as the assay host, were mixed with 5×10^9^ exponentially growing host cells in 10 mL LB with 3-fold replication. The mixture was incubated with orbital shaking for 40 min. Immediately after mixing and every 10 min thereafter, a 1 mL sample from the mixture was centrifuged (RCF  = 1,700×g) for 1 min to pellet the cells then 100 µL from the supernatant was plated on a PP lawn. The attachment rate constant (*k*) is calculated as 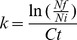



where N_i_ is the total number of phage added, N_f_ is the number of unattached phage, C is the concentration of bacteria, and t is the incubation time in minutes.

### Attachment Protein P3 3D Structure Prediction

Three-dimensional structures of canonical bacteriophage Φ6 ancestral strain P3 attachment protein were predicted by homologue modeling based on nucleotide sequences submitted to the online I-TASSER server (http://zhanglab.ccmb.med.umich.edu/I-TASSER) [21], [22]. I-TASSER generates three-dimensional (3D) atomic models from multiple threading alignments and iterative structural assembly simulations. Default parameters were used for the I-TASSER submission. The P3 amino acid sequence was submitted to the transmembrane structure prediction Dense Alignment Surface software (DAS) website (http://www.sbc.su.se/~miklos/DAS/maindas.html). DAS uses low-stringency dot-plots of the query sequence against a collection of non-homologous membrane proteins using a previously derived, special scoring matrix to identify transmembrane helices of integral membrane proteins. Default parameters were used for the DAS submission.

## Results

### Mutation Frequency

Φ6 HRMs were readily isolated by plating wildtype phages on lawns of the nonpermissive hosts, ERA, TOM and ATRO. The frequency in which ERA-infective HRMs appeared in populations of Φ6 phages was 1.15×10^−6^ (n = 21, SD ±5.219×10^−7^). This value is only slightly lower than Φ6's spontaneous mutation rate, 2.7×10^−6^ [23]. Rates on TOM and ATRO were 1.39×10^−7^ (n = 21, SD ±1.489×10^−8^) and 4.45×10^−7^ (n = 21, SD±1.001×10^−7^) respectively. We conducted a one-way ANOVA on log_10_ mutant frequency with bacterial host strain as a factor, and found significant differences in mutant frequency across different host strains (F = 198.54, DF  = 2, P<0.0001). A Tukey-Kramer post hoc test with α = 0.05 revealed that all compared means were significantly different from each other. Interestingly, HRMs appeared more readily on the phylogenetic outgroup *P. pseudoalcaligenes* ERA than they do on other conspecific *P. syringae* pathovars such as *P. syringae atrofaciens* and *P. syringae tomato* [24].

We found at least 17 unique genotypes among the 69 ERA HRMs isolated and partially sequenced (Table 1). Three HRMs had no mutations in the sequenced region of the genome, thus we count them as, at a minimum, one unique genotype. Out of a combined 78 identified mutations from three studies, the majority resulted in nonsynonymous substitutions in the P3 amino acid sequence. Only 2 synonymous substitutions were identified (Table 1). This result conforms to Duffy et al.'s report of only 1 synonymous substitution among 31 mutations [15]. Synonymous substitution frequencies were similar between the two studies (2.5% vs. 3.2%).

### Mutation Substitution Frequency

Among all nucleotide substitutions identified by our screen, the estimated transition/transversion bias *R* was 1.90. At the 8^th^ residue, at least 5 possible substitutions (G22A, A23G, A23C, G24T and G24C) allow infection of ERA. However, of all substitutions observed, the majority were transitions (51 vs. 9). These results suggest that host range mutations allowing infection of ERA are heavily biased towards transitional substitutions. The significance of this finding is not clear, and may simply be a consequence of spontaneous deamination.

**Figure 1:**
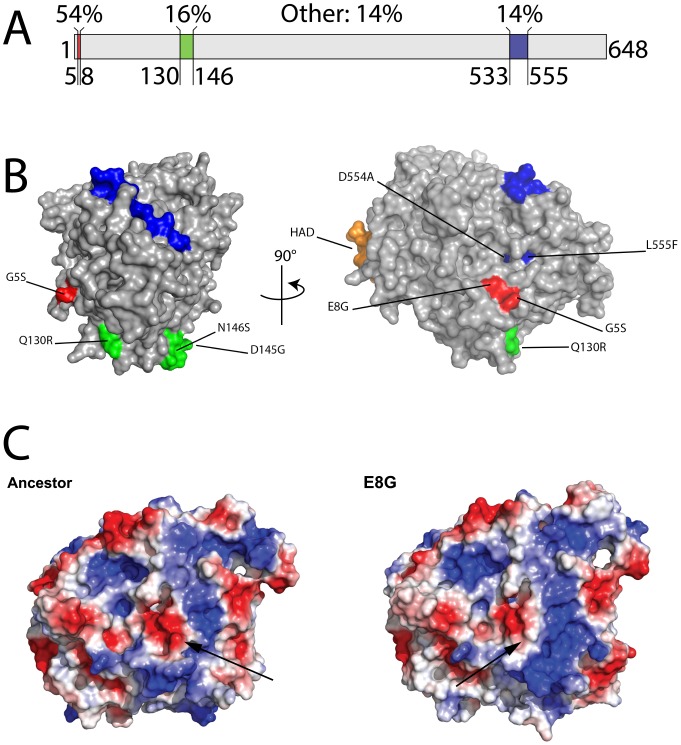
Spatial models of Φ6 P3 protein mutants. Panel A: Three host range mutation hotspots (accounting for 86% of all mutations) are highlighted in this linear representation of the 648 amino acid sequence of the Φ6 P3 gene. The remaining 14% of mutations are not shown. Panel B: Space-filling representations of the Φ6 P3 protein are shown as predicted by I-TASSER. Colored regions correspond to the mutation hotspots depicted in Panel A. A putative hydrophobic anchoring domain (HAD) is shown in orange. In our model, the hydrophobic anchoring domain penetrates Φ6's outer lipid membrane to bind inner membrane protein P6. Panel C: Surface electrical charges of E8G mutant contrasted with ancestor. Space-filling representations showing predicted surface electrical charges for the Φ6 E8G host range mutant and its ancestor were estimated using I-TASSER. Positively- and negatively-charged regions are depicted in blue and red respectively. Arrows indicate the predicted location of the mutated 8^th^ residue. The most prominent difference between the mutant and the ancestor is the greater surface positive charge at the presumed host binding domain.

87% (60 of 69) of all mutants possessed a mutation at the 8^th^ amino acid residue in the P3 protein. Only 9 mutants (4 single, 1 double, 1 triple and 3 unknowns) did not show a mutation at the 8^th^ residue. This imbalance is higher than was observed in Duffy et al.'s study, where only 14 of 30 mutants isolated on ERA possessed mutations at the 8^th^ residue [15]. However, we note that Duffy et al.'s study did not control for the “jackpot effect” and 20 of 30 mutants were isolated from hosts other than ERA. The dominance of a single residue is not unprecedented. Ferris et al. reported that 12 of 40 mutants isolated on *P. glycinea* showed a mutation at the 554^th^ residue [11]. Across all 3 studies and 5 different hosts, there seems to be 3 “hotspots” for host range mutations in Φ6. 85.4% of all amino acid substitutions occurred close to the 8^th^ (54.7%), 138^th^ (16.7%) and 544^th^ (14%) residues (Fig. 1A; Table 2).

**Table 2:** Host Range Substitution Hotspots in φ6 P3 Protein^a^.

Substitution	This Study	Duffy	Ferris	N	Frequency^b^	Combined Frequency^b^
G5S	0	0	2	2	1.3%	54.7%
E8K	23	4	1	28	18.7%	
E8G	28	9	5	42	28%	
E8D	4	0	0	4	2.7%	
E8A	5	1	0	6	4%	
Q130R	7	0	0	7	4.7%	16.7%
A133V	0	9	0	9	6.0%	
D145G	0	0	3	3	2.0%	
N146S	0	0	6	6	3.9%	
D533A	0	0	1	1	0.7%	14.0%
D535N	0	0	1	1	0.7%	
D554G	3	1	8	12	8.0%	
D554A	1	0	1	2	1.3%	
D554V	0	0	1	1	0.7%	
D554N	0	0	2	2	1.3%	
L555F	1	0	1	2	1.3%	
Others	9	6	7	22	14.7%	14.7%
**Total**	**81**	**30**	**39**	**150**	**100%**	**100%**

Amino acid substitutions close together in the primary sequence are grouped together. We combine data from this study with two other studies of φ6 host range expansion. N is total number of times a substitution was observed across all studies. Frequency is percentage of total substitutions a particular substitution was observed. Combined frequency is percentage of total substitutions constituted by substitutions in a particular region of the primary sequence. Others category includes substitutions found outside substitution hotspots.

a Data compiled from this study, Duffy et al. 2006 and Ferris et al. 2007 [11], [15].

b Some frequencies rounded off to nearest tenth percent.

### Phenotypic Change Analysis

Changes in mass, electrical charge and hydrophobicity presumably can alter host receptor binding by changing the protein's tertiary structure and altering protein-protein interactions. In Table 3, we compiled the phenotypic characteristics of all amino acid substitutions allowing infection of ERA observed in this study and in Duffy et al. [15]. Using this data, we performed a paired t-test on amino acid mass for each mutation with strain type (wildtype or mutant) as a factor. Both factors had significant effects on amino acid mass. Substituted amino acids in mutants had significantly less mass than the original amino acids in the wildtype strain (t = 6.73, DF  = 77, P<0.0001). This effect was most pronounced in mutation hotspots (F = 7.25, DF  = 71, P<0.0001). Perhaps lower mass substitutions permit greater flexibility at the host binding site.

**Table 3:** Amino Acid Substitutions Associated with φ6 Host Range Expansion on ERA.

Amino Acid Substitution	TS^a^	Du^b^	N	Average Mass		Electrochemical Properties		Hydrophobicity Index	
				Wildtype	Mutant	Wildtype	Mutant	Wildtype	Mutant
**Glutamic Acid to Lysine (E8K)**	23	4	27	129.1	128.2	Acidic (−) Polar	Basic (+) Polar	−3.5	−3.9
**Glutamic Acid to Glycine (E8G)**	28	9	37	129.1	57.1	Acidic (−) Polar	Neutral Nonpolar	−3.5	−0.4
**Glutamic Acid to Aspartic Acid (E8D)**	4	0	4	129.1	115.1	Acidic (−) Polar	Acidic (−) Polar	−3.5	−3.5
**Glutamic Acid to Alanine (E8A)**	5	1	6	129.1	71.1	Acidic (−) Polar	Neutral Nonpolar	−3.5	1.8
**Aspartic Acid to Alanine (D35A)**	0	2	2	115.1	71.1	Acidic (−) Polar	Neutral Nonpolar	−3.5	1.8
**Phenylalanine to Serine (F46S)**	1	0	1	147.2	87.1	Neutral Nonpolar	Neutral Polar	2.8	−0.8
**Phenylalanine to Leucine (F46L)**	1	0	1	147.2	113.2	Neutral Nonpolar	Neutral Nonpolar	2.8	3.8
**Glutamine to Arginine (Q130R)**	7	0	7	128.1	156.2	Neutral Polar	Basic (+) Polar	−3.5	−4.5
**Alanine to Valine (A133V)**	0	9	9	71.1	99.1	Neutral Nonpolar	Neutral Nonpolar	1.8	4.2
**Serine to Proline (S161P)**	1	0	1	87.1	97.1	Neutral Polar	Neutral Nonpolar	−0.8	−1.6
**Serine to Threonine (S246T)**	0	2	2	87.1	101.0	Neutral Polar	Neutral Polar	−0.8	−0.7
**Serine to Tryptophan (S299W)**	1	0	1	87.1	186.2	Neutral Polar	Neutral Slightly Polar	−0.8	−0.9
**Lysine to Threonine(K311T)**	0	1	1	128.2	101.0	Basic (+) Polar	Neutral Polar	−3.9	−0.7
**Glycine to Serine (G515S)**	1	0	1	57.1	87.1	Neutral Nonpolar	Neutral Polar	−0.4	−0.8
**Aspartic Acid to Glycine (D554G)**	3	1	4	115.1	57.1	Acidic (−) Polar	Neutral Nonpolar	−3.5	−0.4
**Aspartic Acid to Alanine (D554A)**	1	0	1	115.1	71.1	Acidic (−) Polar	Neutral Nonpolar	−3.5	1.8
**Leucine to Phenylalanine (L555F)**	1	0	1	113.1	147.2	Neutral Nonpolar	Neutral nonpolar	3.8	2.8

Amino acid substitutions found to allow φ6 infection of Pseudomonas pseudoalcaligenes ERA from data obtained by two separate studies. N is number of times substitution was observed across the two studies. Average mass is residue weight of the original and substituted amino acid [67]. Hydrophobicity index was obtained from Kyte and Doolittle [68]. Negative numbers are more hydrophilic; positive numbers are more hydrophobic.

a =  This study; ^b^ =  Duffy et al. 2006 [15].

Electrostatic interactions between host and phage proteins are most likely the basis of phage attachment. If so, we expect that charge changes incurred by host range mutations should be consistently in the same direction. A *Χ* ^2^ test was used to determine whether chemical properties of substituted amino acids differed significantly from the random expectation based on the amino acid composition of the P3: 9.16% acidic, 8.69% basic, 24.53% hydrophilic, and 57.45% hydrophobic. We found that mutant amino acids were significantly more likely to be basic or hydrophilic than expected by chance (*Χ* ^2^  = 110.008, DF  = 3, P<0.0001). Furthermore, the frequency of mutations occurring at acidic residues was disproportionately high (81/106 or 76%). Ferris et al. also observed a greater than expected number of loss of charge mutations [11]. We speculate that these chemical changes make the P3 protein's host-binding site more permissive for binding host receptors.

### P3 3D Structure Prediction

Little is known regarding how host range expanding amino acid substitutions affect phage attachment protein structure. We used I-TASSER [21], [22] and DAS modeling software [25] to predict structural features of the P3 protein. I-TASSER generates three-dimensional atomic models from multiple threading alignments and iterative structural assembly simulations based on homology to solved structures (Fig. 1B). The predicted model's confidence score (C-score) was −2.12, which is intermediate confidence where scores range from high (2) to low (−5) confidence. When predicting known structures, and using a C-score cutoff >−1.5 for the models of correct topology, both false positive and false negative rates are below 0.1 [21]. While our C-score did not meet this threshold, we are confident that the probability of an incorrect structure is still low. Our view is supported by the ability of the predicted structure to provide a biologically plausible interpretation of the mechanistic basis of host range expansion.

DAS modeling software predicts transmembrane protein segments based on low-stringency dot-plots of query sequences against a collection of non-homologous membrane proteins using a previously derived, scoring matrix. Although P3 is soluble [26], DAS predicted a 21 amino acid hydrophobic membrane-interactive domain at residues 271 to 291. Based on the fact that, on the predicted structure, this domain extends out from the P3 core (Fig. 1B), we venture that domain likely anchors the P3 protein to the integral membrane protein P6 [27], thus we will refer to it as the hydrophobic anchoring domain, or HAD. All host range mutations occurred on the face opposite the HAD, suggesting that the opposite surface binds the host receptor, and that mutations in this region allow infection of novel hosts. However, this hypothesis assumes that amino acid substitutions do not substantially alter the protein shape, and that residues on this face in the ancestor would remain on this face in the mutant. Figure 1C suggests our conjecture is valid as the E8G mutant's predicted structure does not show major structural rearrangements compared to the wildtype. Interestingly, the most common host range mutations found in our study alter the surface charge at this location from negative to neutral, hinting at a proximate mechanism for host range expansion (Fig. 1C).

### Plaque Size

We isolated HRMs by visually identifying and picking plaques off lawns of the nonpermissive host, ERA. Our results showed that host range mutations were heavily biased towards the 8^th^ residue. One possible criticism of our mutant isolation process is that it may have been biased towards certain mutations simply because these mutants formed larger plaques that were more likely to be spotted by the sampler. To test this hypothesis, we determined from digital photographs the average plaque size for 13 of 17 of our identified HRMs. Mean plaque size for our mutants ranged from 3.5 to 10.3 mm^2^ (Table 4). We performed an ANOVA of mean plaque size with mutant frequency as a factor, and the results confirmed that plaque size did not predict mutant frequency. While we did find significant differences in plaque size among genotypes, the two most frequent genotypes found by our study (E8K and E8G) ranked 4^th^ and 9^th^ respectively in mean plaque size. These data imply that mutant sampling was not biased. Furthermore, we did not observe any correlation between fitness and plaque size.

**Table 4:** Phenotypic Characteristics of Bacteriophage φ6 Mutants.

Strain	Mutation	Frequency of Mutant	Fitness ERA	Fitness PP	Plaque Size ERA^a^	Attachment rate to ERA^b^	Attachment rate to PP
**S8**	D554G	2	3.31	14.73	6.89	2.58×10^−11^	4.79×10^−11^
**S68**	E8D	4	1.97	14.61	5.69	2.22×10^−11^	4.72×10^−11^
**S53**	E8A	4	3.97	14.57	7.8	4.80×10^−11^	4.68×10^−11^
**S46**	Q130R/S299W	1	0.98	14.80	9.26	1.68×10^−11^	5.05×10^−11^
**S42**	S161P/L555F	1	2.20	14.93	9.17	2.40×10^−11^	5.31×10^−11^
**S4**	Q130R	1	1.27	14.74	6.28	2.05×10^−11^	5.41×10^−11^
**S30**	E8K/Q130R	1	2.51	14.62	3.54	2.49×10^−11^	5.71×10^−11^
**S28**	E8A/F46L	1	2.47	15.21	5.94	2.53×10^−11^	5.63×10^−11^
**S26**	D554A	1	4.47	14.77	7.85	2.39×10^−11^	5.63×10^−11^
**S154**	E8G	24	4.12	14.92	6.29	2.59×10^−11^	5.33×10^−11^
**S14**	E8K/D554G	1	2.08	14.79	6.56	2.09×10^−11^	4.50×10^−11^
**S13**	E8K/F46S	1	3.16	14.85	10.25	2.24×10^−11^	4.60×10^−11^
**S117**	E8K	20	3.91	14.66	8.93	2.51×10^−11^	4.39×10^−11^
**φ6 WT**	n/a	n/a	n/a	15.07	n/a	n/a	4.91×10^−11^

a =  in mm^2^; ^b^ =  Attachment rate (*k*) units are per milliliter per cell (or per phage) per minute.

### Mutant Fitness on Original and Novel Hosts

**Figure 2:**
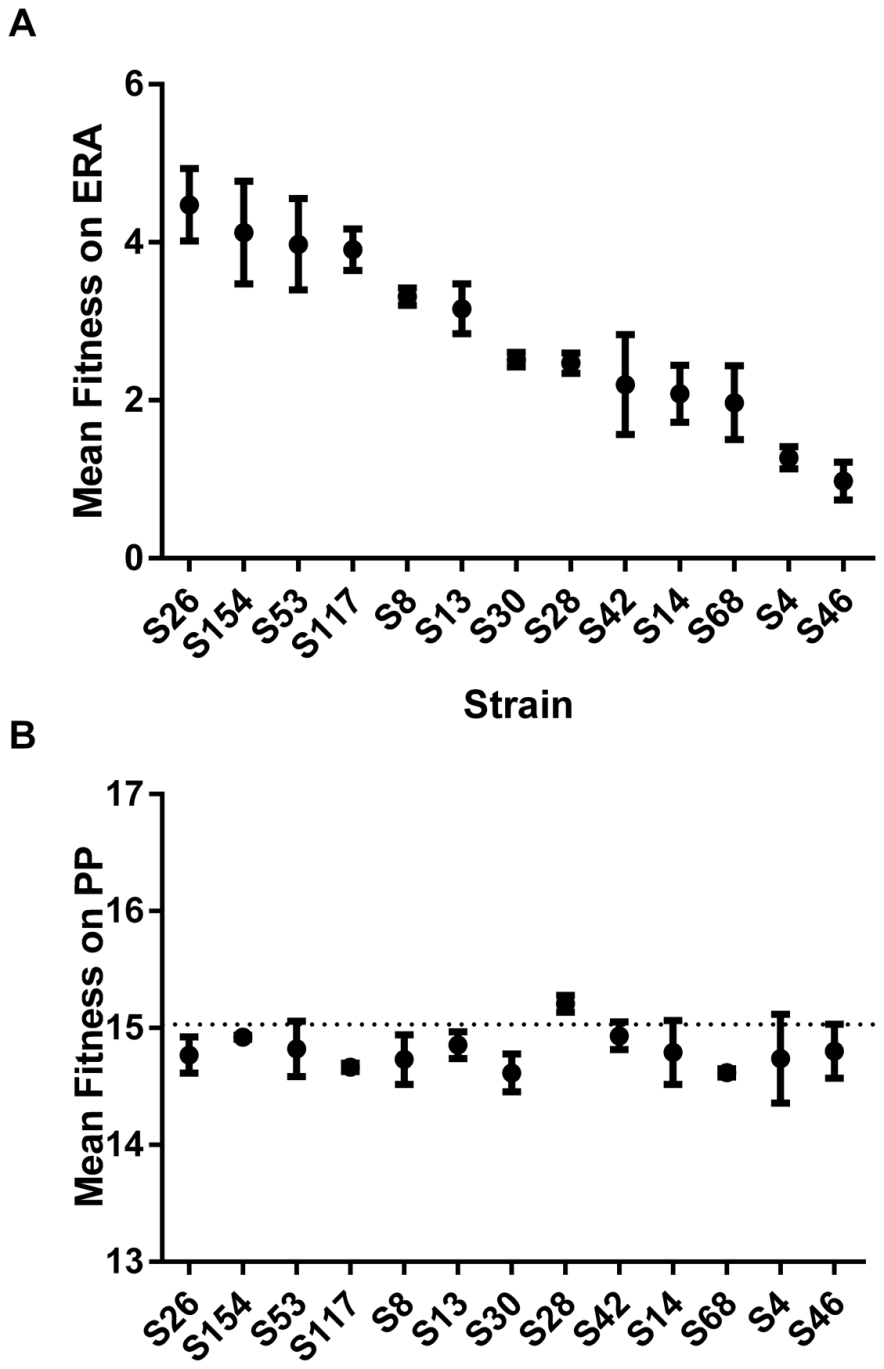
Mutant absolute fitness on canonical and novel hosts. Panel A: Absolute fitness of 13 Φ6 host range mutants on the novel host, ERA. Each point is the mean of 5 replicate measurements of fitness. Bars are ±1SE. Panel B: Absolute fitness of 13 Φ6 host range mutants on the canonical host, PP. Each point is the mean of 5 replicate measurements of fitness. Fitness of wildtype Φ6 is shown by the dotted line for comparison. Bars are ±1SE.

The fitness consequences of host range expanding mutations will play a large role in the ability of these mutants to persist in host populations [28]. With this in mind, we estimated the absolute fitness of 13 of our mutant genotypes on the canonical host, PP, and the novel host, ERA (Table 4). A one-way ANOVA of absolute fitness with strain as a factor revealed significant differences among strain fitness on both ERA (Fig. 2A; F = 40.64, DF  = 12, P<0.0001) and PP (Fig. 2B; F = 3.515, DF  = 12, P = 0.0008), but mean fitness on ERA was not correlated with mean fitness on PP nor was fitness on ERA correlated with the number of mutations a mutant possessed. In fact, genotypes containing multiple mutations tended to be less fit than those with single mutations, although this trend was not significant. Matching previous results, fitness on PP was, in all but one case, less than that of the ancestor [11], [15]. These results are indicative of antagonistic pleiotropy, implying a tradeoff in fitness between infection of PP and ERA. In addition, the coefficient of variation (i.e. standard deviation/mean; CV) in mutant fitness was considerably greater on ERA as opposed to PP (CV: 0.402 versus 0.015). This suggests that mutations expanding the host range have a much wider range of fitness effects on the novel host.

### Attachment Rate

**Figure 3:**
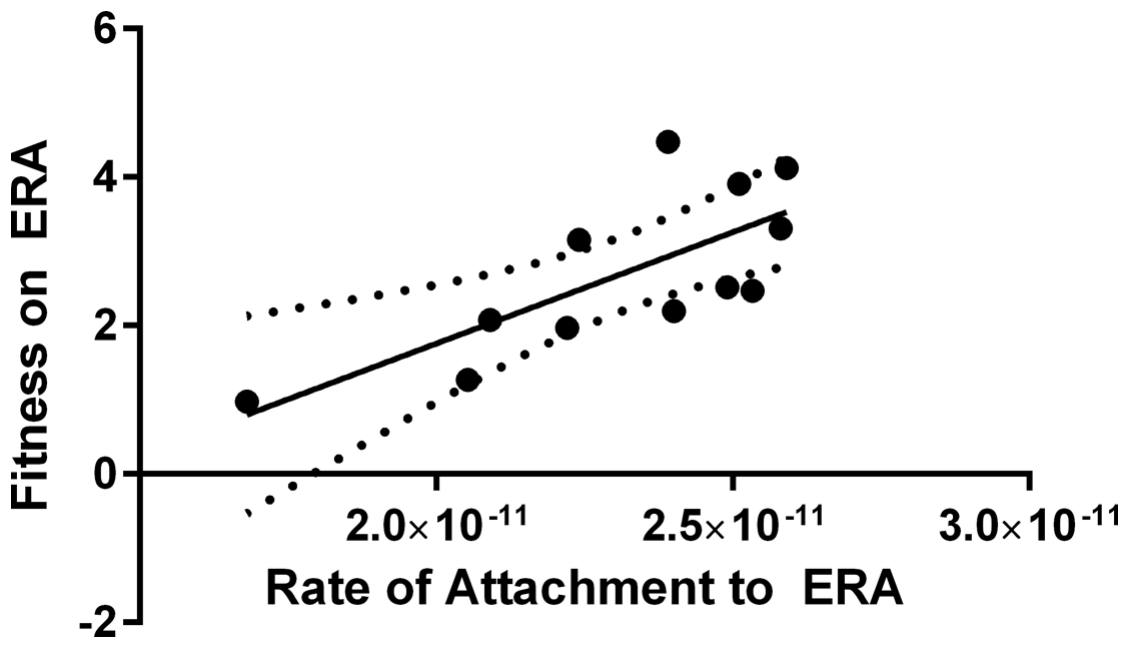
Mean ERA attachment rate (k) is plotted against phage Φ6 fitness on ERA. Attachment to ERA was correlated with fitness on ERA for Φ6 host range mutants. Each point is the mean of 3 replicate measurements. Dotted lines show 95% confidence intervals.

Bacteriophages initiate infections of host cells by binding to receptors on the surface of the bacterial outer membrane. As such, the host attachment rate is a critical factor in the ecological success of a phage. We measured the rate of phage attachment to the original and novel hosts for 13 mutant genotypes (Table 4). A one-way ANOVA of the rate of attachment to ERA with mutant genotype as a factor revealed significant differences among the strains (F = 10.17, DF  = 12, P<0.0001). In addition, we regressed attachment rate against mutant fitness on ERA to determine if the two were correlated. Since our HRMs most likely differ only by mutations in the P3 host attachment protein, we expected that improved attachment would lead to increased fitness. Indeed, for 13 mutant strains whose fitnesses and attachment rates were estimated, fitnesses on ERA were correlated with ERA attachment rates (Fig. 3; F = 11.91, DF  = 1, P = 0.0062). However, the linear regression model accounted for roughly half of the variance in attachment rate (R^2^ = 0.54). These results are not surprising given the difficulty of precisely estimating the Φ6 attachment rate. Nevertheless, the results conform to our expectation of a positive correlation between fitness and host binding ability. By contrast, attachment rates of the various mutants to PP were not significantly different, nor were they correlated with mutant fitnesses on this host. These results might be expected given the relatively narrow range of fitness differences on PP (Fig. 2; Table 4).

The rates of attachment to PP were significantly greater than attachment rates to ERA (One-way ANOVA: F = 216.7, DF  = 1, P<0.0001). This latter result matches expectations since mutant fitness on PP is approximately an order of magnitude greater than that on ERA [17]. Presumably, the switching of receptor types and the lack of adaptation to an ERA receptor may account for the significant differences in mutant fitness on the different host types. However, attachment rates to PP and ERA were not correlated, implying that mutations that increase binding to ERA do not necessarily increase or decrease binding to PP.

## Discussion

### Φ6 Host Range Mutation Frequency

Understanding the genetic basis of virus host range expansion is critical to predicting the emergence of potentially dangerous viruses. The genetic distance a virus must cross to gain the ability to infect a novel host may be a dominant factor determining the probability of emergence. Not all viruses readily infect novel hosts [29]. For example, many mycobacteriophages isolated on *Mycobacterium smegmatis* are unable to infect *M. tuberculosis*, even when large numbers of phage are plated [30]. Presumably infection of *M. tuberculosis* requires several simultaneous mutations or even the recombination of whole genes or gene systems. By contrast, many viruses are able to infect novel hosts via single nucleotide substitutions [10], [31]–[36]. This minimal genetic distance can easily be traversed because viral population sizes and mutation rates allow them to search available sequence space rapidly. The phage Φ6 is an excellent model to study virus emergence via single nucleotide substitutions because such HRMs are easily isolated, sequenced, and characterized in the laboratory [11], [15].

In this study, we found that Φ6 HRMs appear on ERA at a rate (1.17×10^−6^) slightly lower than the estimated Φ6 mutation rate of 2.7×10^−6^ per nucleotide per generation. Thus our figure seems somewhat low given that there are multiple possible mutations allowing host range expansion in the Φ6 genome (Table 1). However, Chao et al.'s estimate was derived from the frequency of revertants from an amber mutation (*sus*297), and it was assumed that there was only one way to revert [23]. If there are multiple ways to revert from Chao's et al.'s amber mutation, then theirs is an overestimate of the mutation rate. Moreover, Chao et al. estimated the mutation rate at a single locus, but the mutation rate may vary across the genome [37], [38]. At any rate, it is clear that, given their potentially enormous population sizes, Φ6 HRMs can be isolated relatively easily.

Our results indicate that there is considerable variation in the ability of Φ6 to mutate to infect nonpermissive host strains. While there are certainly strong coarse-grained trends in infectivity, e.g., Φ6 seems mainly restricted to the pseudomonads [39], infectivity within this group is currently unpredictable. Phage Φ6 is better able to mutate to infect *P. pseudoalcaligenes* ERA, a distant relative of *P. syringae* pv *phaseolicola* [24], than two pathovars from the same species, *P. syringae* pv *tomato* and *P. syringae* pv *atrofaciens* [40]. Duffy et al. and Cuppels et al. found many examples of other *P.syringae* pathovars nonpermissive for Φ6 even at high plating densities [15], [39]. For example, Duffy et al. were unable to isolate HRMs on at least 8 *P. syringae* pathovars despite plating over 10^10^ Φ6 phages on each pathovar [15]. Similar results were obtained by Cuppels et al. [39]. It would appear that phylogeny is a poor predictor of infectivity, at least at the fine scale level within the pseudomonads. Φ6's ability to expand its host range appears to be somewhat idiosyncratic, which is to be expected given myriad possible outcomes for parasite-host coevolution [41]. It may be that the *P. syringae* strains have experienced recent coevolution with Φ6 or its close relatives, and thus have acquired resistance to infection to these phages. By contrast, more distantly Pseudomonads may not have recently experienced consistent Φ6 infection, therefore remain relatively sensitive to this virus.

The frequency of mutants lacking mutations in the P3 (4.3%) was similar to that found in Ferris et al.'s study (2.5%) [11]. These results provide strong evidence that the P3 sequence is the primary, but not exclusive, determinant of host range among phage Φ6 [42]. While it is tempting to speculate that additional host range mutations might be found in membrane fusion protein P6, Duffy et al. sequenced the P6 for 30 Φ6 HRMs and found no mutations [15]. As of publication, no other candidate genes for host range expansion on ERA have been explicitly identified in Φ6; however one study has reported that a mutation allowing infection of ERA was localized to the large segment [43]. This segment contains a gene encoding an RNA-dependent RNA polymerase and genes associated with RNA packaging and procapsid assembly [12].

The number of ways a virus can mutate to infect a novel host is an important parameter in predicting its potential for emergence [28]. Using a method based on the coupon collector's problem of statistical theory, Ferris et al. estimated the total number of possible mutations that allow Φ6 to infect a novel host, *P. glycinea* [11]. The coupon collector's problem can be informally stated as: Given *n* coupons, how many coupons will need to be sampled before each coupon is observed at least once [44]? One assumption of the coupon collector's problem is that all coupons are equally likely. This assumption does not hold for genetic mutations as some types are more likely than others are. Ferris et al. accommodate this simplification by adjusting the equation to account for differences in the probabilities of transitions and transversions. Since they found 19 distinct genotypes among their 40 independent samples, they estimated that further sampling would uncover an additional 36 mutations [11]. If Ferris et al.'s estimates are correct, it would mean that 1.3% of all possible nonsynonymous substitutions in P3 confer the ability to infect ERA (i.e., 55 of 4,380 potential nonsynonymous changes expand host range).

Although their HRMs were isolated on a different host, *P. glycinea*, both their study and ours found similar frequencies of transitions among all mutations (90% in Ferris et al., 84% in our study). However, out of 69 HRMs, we found only 17 distinct genotypes. Ferris et al. isolated almost the same number of distinct genotypes in half as many samples [11], which may be a consequence of the different hosts of isolation. Since Duffy et al. observed 10 unique genotypes out of 30 isolates (33% unique) [15] and we observed at least 17 unique genotypes out of 69 (26% unique), the implication is that more unique genotypes would be found with further sampling. However, a closer inspection of our data suggests otherwise. 8 of 17 of our unique genotypes were only unique because of second- or third-site mutations. If we consider only those mutations that are *sensu stricto* necessary for infection of ERA, we only find a combined 13/99 (13%) unique genotypes among our and Duffy et al.'s study [15]. In fact, we only found 3 unique *sensu stricto* substitutions not found by Duffy et al. study and they found 6 not identified in ours.

If 1.3% of all possible nonsynonymous substitutions allowed Φ6 to infect ERA, we would expect to see more unique genotypes among our isolates. Our results also indicate that some mutations occur far more frequently than expected by chance even if differences in transitions and transversions are accounted for. One possibility is that low fitness HRMs are eliminated by within plaque selection and consequently are not represented in the mutant collection sampled. We have no means to ascertain the validity of this hypothesis at this point, but it could be an interesting question to approach by deep sequencing of single HRM plaques. However, at the same time, it seems likely that additional factors that are not currently well understood, such as RNA structure, codon bias and variation in the mutation rate across the genome, influence the probability of mutation at any particular locus. Nonetheless, Ferris et al.'s method is a valuable step forward towards the estimation of an important parameter relevant to virus emergence.

### Mutation Hotspots

We found that mutations expanding the host range of phage Φ6 were more likely to appear in certain regions of the P3 gene than others. Such mutation hotspots have been observed among virus drug resistance [45]–[47], host range [48], [49], hemagglutinin [50], capsid [51], and core antigen genes [52] among others. Mutation hotspots are evidence of strong positive selection for substitutions that provide an adaptive advantage in a particular environment [53], [54]. Growth on a novel host should impose strong positive selection for nonsynonymous substitutions at loci associated with host range expansion. Thus, we can use the frequency of mutations found in our survey to identify regions of the P3 protein that are important in attachment to a host receptor. 85.4% of all mutations identified by our study and by Duffy et al. [15] were found in just three regions (near 8^th^, 133^rd^ and 554^th^ residues) of the P3 gene (Fig. 1A; Table 2). We venture that these hotspots on the P3 protein are important in host range determination among Φ6 phages.

### Structural Speculations

We used the structural modeling software I-TASSER [21], [22] to predict the structure of the P3 protein from its amino acid sequence. The resulting structure showed homology to bacterial alcohol dehydrogenase quinoproteins [55]–[57]. Interestingly, in the best-fit model, our putative mutation hotspots were located close together on one face of the ∼60 Å diameter P3 protein (Fig. 1B). Residues 8 and 130 were located at the surface 18 Å from each other, and residue 554 was located subsurface about 15 Å from residue 8 and 23 Å from residue 130. Other less frequently observed mutations also occur near this region (Fig. 1B). We propose that this region of the P3 protein is a host-binding domain and directly interacts with host receptors. This supposition is supported by the fact that the host binding domain is diametrically opposite the hydrophobic anchoring domain (residues 271–291) predicted by DAS (Fig. 1B). The most parsimonious explanation is that this domain serves to anchor the P3 to the integral membrane protein P6 [27], which leaves the putative host binding domain exposed to the environment.

Mutations allowing infection of ERA may not significantly alter the tertiary structure of the P3 protein. I-TASSER structural modeling did not show any major structural rearrangements in predicted structures for mutant strains. Rather mutations may alter the host-binding domain's electrical charge from negative to positive or neutral (Fig. 1C). This difference in electrical charge may allow mutant Φ6 to bind the ERA host receptor. The presumptive ERA receptor is its pilus, but this has not been definitively determined. If the ERA receptor were indeed the pilus, it would be interesting to know if its electrical properties are appreciably different from those of the pilus of PP. Moreover, it is plausible that neutral or positive electric charges and smaller mass amino acids confer more flexibility to the binding region, allowing a greater variety of structures to be bound [58]. It would be interesting to determine if host range expanding mutations more frequently result in the substitution of small for large amino acids or alter the charge of the binding site.

### Fitness on Native and Novel Hosts

Fitness on native and novel hosts was assessed using standard flask productivity assays. Phage Φ6 HRMs showed a broad range of fitness values on ERA, some of which were significantly different from the others (Fig. 2A). Mutant fitnesses on the native host, PP, were much greater than those on ERA (Fig. 2B). Since Φ6 is presumably well adapted to native but not novel hosts, these results meet our expectations. Supporting these results, we found that the coefficient of variation (CV) of mutant fitnesses on PP was much lower than CV of mutant fitnesses on ERA. These results conform to theoretical expectations that there should be less variation in fitness values close to a fitness peak on an adaptive landscape [59]. Directional selection should erode the variation in fitness as a population increases in fitness in a particular environment. Thus, a virus that is adapted to a particular host should have lower variation in fitness on that host as opposed to a host to which it is not well adapted.

We found that, in concert with previous studies [11], [15], mutations expanding the Φ6 host range usually reduced fitness on the original host, PP. On average, HRM fitness on PP was reduced about 2.5% compared to the wildtype. Negative genetic associations between host types is an example of antagonistic pleiotropy [60], [61]. The adage that “a jack of all trades is a master of none” is well supported, at least among Φ6 host infections. However, the ultimate cause of host specialism or generalism remains opaque. Intuitively one would imagine that a broader host range would produce greater returns than a narrow one as long as the reduction in productivity on a single host was offset by an increase in overall productivity [62]. With regard to the present system, it seems unlikely that the relatively minor cost in fitness on the original host imposed by host range expansion should outweigh the benefits of an expanded host range. Moreover, we isolated one mutant (S28) whose fitness on the canonical host actually increased following the acquisition of a mutation permitting infection of ERA. Why then are broad host range phages relatively rare? The rarity of generalism may be a result of the interaction of widespread habitat patchiness, reduced dispersal and the ubiquity of local adaptation [63]. If these general trends hold, competition within a patch should favor the evolution of specialism. This hypothesis should be amenable to testing via experimental evolution studies.

As a rule, we might expect that novel hosts will present a greater challenge to virus reproduction than native hosts, a conclusion that is supported by many examples in the literature [64]–[66]. Novel hosts may represent ecological sinks, defined as habitats where the basic reproductive rate is <1. Our fitness results support this conjecture, and suggest that Φ6 probably experiences a broader range of sink conditions on ERA than it does on PP. Consequently, Φ6 population extinction is more likely in a habitat populated by ERA than one populated by PP [17]. Given the many HRM genotypes over a broad range of fitness values, Φ6 should be a valuable system to test hypotheses regarding virus emergence [28].

### Attachment to Native and Novel Hosts

With the exception of the three non-P3 mutants, the mutant strains are most likely isogenic outside the host attachment protein region. The differences in fitness are expected to result mainly from differences in binding efficiency to the host receptor. Our results indicate that different suites of mutations had highly divergent attachment rates and fitnesses on the novel host (Fig. 2 and 3). Nonetheless, a regression of phage fitness on ERA against attachment rate to ERA revealed a significant positive correlation. Ferris et al. reported a similar result for Φ6 infecting *P. glycinea* [11]. These results make intuitive sense as mutants that are better able to bind to the host are expected to reproduce at a higher rate. Moreover, attachment to ERA was significantly lower than to PP, which is also reflected in the large differences in fitness.

### Implications for Disease Emergence

This study and other recent studies of Φ6 host range expansion suggest several generalizations. First, phylogeny may only allow relatively coarse-grained predictions of virus host range. Phage Φ6's ability to mutate to infect close relatives was frequently worse than its ability to infect distant relatives. Second, nonsynonymous substitutions allowing host range expansion may occur at hotspots in the host attachment protein. This prediction makes intuitive sense as host attachment relies on binding affinity between host and virus proteins. In addition, many host range-expanding mutations may not result in large structural rearrangements in host attachment proteins. Rather, amino acid substitutions may result in more subtle changes in protein surface charges, allowing binding to different host proteins. Furthermore, the number of nonsynonymous substitutions allowing host range expansion is probably relatively small considering the number of possible substitutions. Nonetheless, the relatively high virus mutation rate allows viruses to rapidly acquire host range expanding mutations despite their relative rarity. Finally, initial fitness on a novel host is usually much less than that on the original host, and antagonistic pleiotropy among host range mutations is common. This generalization conforms to our expectations since evolutionary tradeoffs in different habitats are anticipated to be ubiquitous.
